# Exploring ways to improve healthcare service access for people experiencing homelessness in Manchester, UK

**DOI:** 10.1093/heapro/daaf108

**Published:** 2025-07-10

**Authors:** Alya Howard, Amanda Low, Natasha Howard

**Affiliations:** Faculty of Biology, Medicine and Health, University of Manchester, Oxford Road, Manchester M13 9PL, United Kingdom; Saw Swee Hock School of Public Health, National University of Singapore and National University Health System, 12 Science Drive 2, Singapore 117549, Singapore; Saw Swee Hock School of Public Health, National University of Singapore and National University Health System, 12 Science Drive 2, Singapore 117549, Singapore; Department of Global Health and Development, London School of Hygiene & Tropical Medicine, 15-17 Tavistock Place, London WC1H 9SH, United Kingdom

**Keywords:** homelessness, quality of care, healthcare access

## Abstract

Homelessness is a significant social issue in the UK, affecting the health and life chances of ∼320 000 people annually. This study aims to explore primary healthcare provision from the perspectives of people experiencing homelessness (PEH) in the Greater Manchester area. We conducted a qualitative multimethod study, including unstructured observations and semistructured interviews with 20 PEH across four homelessness day facilities in Greater Manchester during April–May 2023 and analysed data thematically using inductive coding. We generated five inductive themes consisting of PEH fears around communication, challenges navigating the health system, insufficient service signposting, travel as a barrier to healthcare access, and the crucial importance of outreach. The findings indicate that general practitioners can improve communication approaches, clarify pathways to care for PEH, and increase outreach services where feasible to help ensure PEH are better able to access needed services.

Contribution to Health Promotion

- Emerging themes corroborate and supplement existing literature about factors to consider when planning and providing health services for people experiencing homelessness (PEH).
- Health services that catered to and/or addressed challenges specific to PEH were particularly well-received, e.g. outreach van services.
- Additional insights reveal nonhealth factors that influence their decision to visit healthcare facilities, e.g. availability of technological infrastructure such as Wi-Fi.
- Considering the quality of care received and broader circumstances and lives of PEH is essential to improving their healthcare access and utilization, as health is often seen as a competing priority amongst other things necessary for their survival.

## INTRODUCTION

### Homelessness and health

Experiencing homelessness is strongly associated with poorer health outcomes. When compared with the housed population, people experiencing homelessness (PEH) face significantly higher rates of acute and chronic diseases, mental health conditions, substance misuse, and trauma, alongside higher morbidity and mortality rates (Aldridge *et al*. 2019). PEH often contend with ‘tri-morbidity’—the simultaneous presence of physical illness, mental illness, and substance misuse—leading to complex healthcare needs (Elwell-Sutton *et al*. 2017). Common conditions such as infectious diseases, injuries, and trauma are exacerbated by low adherence to medical treatment (Bedmar *et al*. 2022). Notably, PEH may have life expectancies that are substantially lower than the general population, with mortality rates two to five times higher (Aldridge *et al*. 2019, Crisis 2023).

### Homelessness in the UK

Approximately 320 000 people are estimated to be homeless or at risk of homelessness in the UK, although actual figures are likely much higher due to underreporting and varying measurement systems (Berry 2021, Crisis 2023). Three categories of homelessness recognized in the UK are rough sleeping (i.e. sleeping on the streets); statutory homelessness (i.e. lacking secure housing or at risk of becoming homeless and legally entitled to help from local authorities to secure accommodation and including individuals who are unintentionally homeless (e.g. due to domestic violence or financial crisis) or in a ‘priority need’ category (House of Commons Library 2024); and hidden homelessness (i.e. individuals either not entitled to or not having requested local authority help with housing, which includes those staying in ‘concealed’ housing such as squats or friends’ sofas) (Crisis 2023). Homelessness can be persistent, temporary, or intermittent and reflects broader societal issues, including poverty, lack of affordable housing, and insufficient support for vulnerable populations (Aldridge 2020, Becker and Foli 2022, Crisis 2023). Homelessness also exacerbates social isolation and exclusion, disproportionately affecting health equity among marginalized groups (Clapham 2007). Recognizing the stigmatizing impact of language, we refer to those affected as ‘people experiencing homelessness’ (Endeavors 2020). It should be understood as both a health and social issue, shaped by structural determinants such as housing policies, welfare system, and healthcare access (Stafford and Wood 2017, Crear-Perry *et al*. 2021).

### The NHS and PEH access to healthcare

The UK National Health System (NHS), established to address socioeconomic consequences of World Wars I and II by providing free universal healthcare, faces significant challenges meeting PEH needs (Hyde *et al*. 2016). Some research indicates that PEH could be 40 times less likely than housed individuals to be registered with a general practitioner (GP), limiting their access to trusted healthcare professionals and continuity of care (Elwell-Sutton *et al*. 2017, Gunner *et al*. 2019). Transportation and mobility challenges further hinder access to healthcare services (Syed *et al*. 2013). PEH not registered with primary care services are less likely to access secondary care, with rough sleepers—the most severe form of homelessness—experiencing the greatest barriers to primary care access (Rafighi *et al*. 2016, Elwell-Sutton *et al*. 2017, O’Carroll and Wainwright 2019). Despite national healthcare guidelines stating that PEH should not be refused registration and can receive healthcare without a documented address, research suggests that they may experience difficulties registering with a GP due to the transient nature of their living arrangements and a lack of fixed address (BMA 2024, CQC 2024, RCGP 2024).

Changing political and economic circumstances in recent decades have tested the promises on which the NHS was founded. Shifting priorities and dominating ideologies that accompany political and electoral cycles have, in turn, shaped NHS reforms through the years (Ham 2007, Klein 2010, Hyde *et al*. 2016). Austerity measures, often entailing reduced public expenditure during economic downturns, are primarily regressive (Stuckler *et al*. 2017). These may disproportionately affect individuals from lower socioeconomic groups, impacting health status and services access of PEH both directly and indirectly (Stuckler *et al*. 2017).

Reduced spendings on healthcare limits resources channelled into a publicly funded system and places a larger burden on health workers to meet increasing demands for services with fewer resources (Hyde *et al*. 2016, Hernandez 2021). Additionally, reduced welfare spending translates into cuts to social protection funding and programmes and places a greater number of people at risk of becoming homeless (Stuckler *et al*. 2017). Against the backdrop of the compounding effects of austerity measures and cyclical healthcare reforms, healthcare access and provision for PEH have been fragmented and the quality of care delivered has been compromised (Hyde *et al*. 2016, Batchelor and Kingsland 2020, Hernandez 2021).

To improve the health outcomes of PEH, it is crucial to account for how PEH use healthcare and how they perceive the quality of the care they receive. The degree of satisfaction that patients have with their healthcare can have a demonstrable impact on the technical quality of healthcare and health outcomes (Escarce and Kapur 2006). ‘Accessible and timely’ healthcare that demonstrates ‘dignity and respect’ and allows ‘choice and control’ are some of the core characteristics of healthcare services that PEH value (Miller *et al*. 2024). A holistic consideration of PEH life circumstances and the characteristics they value in healthcare services aligns with their patterns of healthcare use (McCabe *et al*. 2001, O’Carroll and Wainwright 2019). PEH perspectives can raise important considerations for policymakers and healthcare service providers in terms of issues on which to focus and potential gaps between healthcare provider and patient priorities (Ross *et al*. 2014, Webb *et al*. 2020). Prior studies mostly analysed macro-level organizational reforms and the experiences of health workers within the NHS (Kelliher and Parry 2015, Hyde *et al*. 2016, Batchelor and Kingsland 2020, Bhat *et al*. 2022). Primary research on patient perspectives and experiences of healthcare access for PEH is limited, of which none situates homelessness within broader structural and social determinants of health (SSDH) (Rae and Rees 2015, Gunner *et al*. 2019, Clark *et al*. 2020, Paudyal *et al*. 2020, McConalogue *et al*. 2021). It is thus necessary to examine the healthcare preferences, behaviours, and experiences of PEH to better inform health policies and services (Ahmed *et al*. 2014).

### Aim

We aim to explore PEH perspectives of primary healthcare provision and their interactions with NHS staff and services in Greater Manchester. While many factors contribute to overall PEH well-being, this study focuses on primary healthcare, typically their first point of contact with the NHS (Campbell *et al*. 2015).

## MATERIALS AND METHODS

### Study design

We used an exploratory qualitative multimethod study design, using data from unstructured observations and semistructured interviews with PEH attending homelessness day facilities in Greater Manchester, to enable an interpretivist analysis of barriers to care-seeking and experiences of services. Our stance was informed by critical theory and social justice literature (Lipsky 2010, Zacka 2017, Inglis and Christopher 2018). As such, we recognize that homelessness arises from broader structural and social forces rather than individual weaknesses. We acknowledge our role in data co-production and therefore amplify the voices and experiences of PEH to contest dominant narratives and contribute to broader social policy discussions on reducing homelessness as a determinant of health. Our analysis emphasized how power dynamics, social inequalities, and societal structures perpetuate homelessness.

### Study sites

We included four homelessness day facilities in the Greater Manchester area, anonymized as Facility 1–4. All facilities offered hot food and beverages, internet and telephone access, social support, and advice for PEH. Facility-2 additionally offered discounted toiletries, clothes, and furniture for any clientele on government benefits. Facility-3 additionally offered shower and laundry services, with a free clothes bank. Three were located within 10–25 min’ walk of a dedicated ‘PEH-friendly’ GP clinic (also anonymized), while Facility-4 was a 45-min walk away. This GP clinic, while also offering standard primary care services to domiciled patients, catered to PEH and positioned itself as ‘PEH-friendly’ by intentionally providing a welcoming environment and maintaining dedicated drug and alcohol support teams, podiatrists, sexual health/contraceptive services for sex workers, and an outreach van that regularly visited homelessness day facilities within its catchment.

### Participant sampling and recruitment

We used purposive sampling for maximum variation in terms of sex, age, type of homelessness (e.g. rough sleeping, statutory, hidden), and whether born in or outside the UK (e.g. to include the perspectives of asylum seekers and refugees) (Patton 2014). To obtain a range of experiences of healthcare-seeking and perspectives, we recruited potential participants in person from among PEH attending one of the four homelessness day facilities. Eligibility criteria for participants included being an adult (i.e. aged 18 years or above) PEH in the Greater Manchester area irrespective of whether they were current GP patients.

### Consent process

A.H. obtained study consent from facility managers at all four sites and verbal informed consent from each participant before interview, by explaining study aims and processes, answering any questions, and ensuring participants knew they could skip any question or quit at any time with no consequences. We did not collect written consent as facility managers indicated this might be a barrier to participation.

### Data collection

#### Semistructured interviews

We developed an interview guide, with feedback from GPs working with this population, to capture key challenges experienced by PEH attempting to access health services. The guide was semistructured, enabling coverage of specific topics (e.g. demographic characteristics such as age, gender, length of homelessness, whether born in the UK or not) while allowing for deviation and anecdotal evidence.

A.H. conducted face-to-face semistructured interviews in homeless facilities over five weeks in April–May 2023. After obtaining verbal informed consent, A.H. asked participants if they wanted to move to a quiet room within the facility to conduct the interview. Each interview was conducted in English and took ∼15–35 min, with detailed field notes taken, as facility managers did not provide approval for audio recording. Minimal sociodemographic data were collected, and personal information and identifiers were removed from field notes to ensure anonymity and confidentiality.

#### Unstructured observations

A.H. conducted observations of facility waiting areas, including ‘PEH-friendly’ GP clinic signage and advertising and staff engagement with service users at each site, after obtaining facility managers’ consent and following methodological guidance described in Morgan *et al.* (2017) and Walshe *et al.* (2012). A.H. took digital photographic records of relevant phenomena (e.g. signage) using an iPhone and written records of activities and interactions within the facility waiting area using pen and paper. To maintain confidentiality, A.H. took no photographs of people or personal items, and written records were all de-identified.

### Analysis

A.H. analysed fieldnote data using reflexive thematic analysis as described by Braun and Clarke (2021). This consisted of data familiarization, generating initial codes, searching for themes, reviewing and defining theme names, and writing up the analytic narrative and participant quotes with inputs from N.H. A.L. and N.H. helped refine interpretation and themes.

### Reflexivity

Using Green and Thorogood’s (2018) two-level reflexivity, we situate the research within the UK’s NHS, in terms of focusing on access to healthcare for a marginalized and potentially underserved population, and the NHS’s intersection with the social care system and third sector in terms of access and quality of care for potential NHS service users at specialist homelessness facilities. This intersection provided opportunities for a deeper consideration of SSDH (Luchenski *et al*. 2018). A.H.’s reflexivity follows Abimbola’s framework of declaring ‘gaze’ and ‘pose’ (Luchenski *et al*. 2018, Abimbola 2019). The study contributed to A.H.’s medical degree requirements at the University of Manchester and was primarily intended for a clinical audience. A.H. approached this work as a British female third-year medical student with limited social science research experience, while A.L. and N.H. are experienced public health social scientists based in Singapore who supervised A.H.’s global health research internship.

### Ethics

The National University of Singapore Institutional Review Board (reference NUS-IRB-2024-316) provided institutional ethics approval, with local study approval provided by UVMP Manchester (reference L1AH-2023).

### Findings

#### Participant characteristics

Of 20 PEH interviewed (Table 1), 17 were men and 3 were women, ranging in age from 29 to 63 (mean age 46 years). Participants had experienced unstable living situations ranging from 1 month to 7 years, with an average of 2 years. Fourteen participants were UK-born and six were born outside the country. This demographic range enabled the exploration of different perspectives and experiences of the UK health system, despite the relatively small qualitative sample.

**Table 1.** Participant locations.

Facility	Type	Number interviewed
Facility-1	Homelessness day facility	7
Facility-2	Homelessness community facility	6
Facility-3	Faith-based charity and homelessness day facility	5
Facility-4	Faith-based day facility	2

### Thematic findings

We generated three inductive themes: (i) fears around communication; (ii) difficulties navigating the health system; and (iii) more signposting and outreach to improve healthcare access.

### Fears around communication

We generated two subthemes related to communication fears: (i) language challenges for migrant PEH; and (ii) felt PEH disempowerment.

#### Language challenges for migrant PEH

All six participants born outside the UK expressed fear in accessing UK health services, worsened by embarrassment about their English skills. All expressed the desire for an interpreter if they were to access a GP.

I’m no good at English. What can we say to doctors? (31-year-old male)

I don’t feel like they understand me… I am embarrassed. (32-year-old male)

#### Felt PEH disempowerment

All participants, particularly men, expressed concerns about their ability to advocate for themselves adequately, expressing a general feeling of disempowerment regarding communicating their medical concerns, finding medical terminology intimidating, and clinical environments uncomfortable. They noted that day facility staff often helped service users book their medical appointments, as many participants did not feel sufficiently confident to do this themselves.

I don’t understand all those medical words, and everyone always speaks so quickly… It makes me feel stupid. (56-year-old male)

### Difficulties navigating the health system

We identified four subthemes related to system navigation difficulties: (i) health system complexity and negative ‘gatekeeping’ experiences; (ii) healthcare as competing priority; (iii) substance-use triggers; and (iv) technological barriers in accessing healthcare.

#### Health system complexity and negative ‘gatekeeping’ experiences

Several participants, born in and outside the UK, described the NHS as too complex to navigate. Some noted that waiting times for appointments could be so long that it was not worth seeking their services. Two complained about having to wait over an hour on each visit to the ‘PEH-friendly’ GP clinic and do not consider it a good use of time. Several described negative experiences of trying to access NHS care, such as feeling judged or not listened to by ‘gatekeepers’ (e.g. receptionists).

I can never talk directly to the Doctor. We always have to speak to a receptionist first. (32-year-old male)

The receptionist (at ‘PEH-friendly’ GP clinic) was so rude it put me off. (63-year-old female)

Why should we have to tell a receptionist, who doesn’t have any medical experience, what’s wrong with me? (63-year-old female)

Everyone’s at the mercy of how competent their GP is. (56-year-old male)

Many of these negative perspectives were raised by participants born outside the UK. Among white-English participants, all but one either did not mention difficulties with receptionists or praised them. This further indicated that language difficulties and perceived identity could be significant barriers to healthcare access for migrants.

The receptionist always makes me feel really comfortable […]. She’s very friendly. (56-year-old male)

#### Healthcare as competing priority

Most participants expressed negative emotions about seeking healthcare, claiming it was too much effort and not their top priority.

If people have got housing problems, they’ve already got enough problems to begin with. (56-year-old male)

While participants recognized that their health was important, when combined with the perceived difficulties of getting adequate care, they often consciously deprioritized it, as finding places to sleep and dealing with daily living crises were deemed more urgent priorities.

As a homeless person, we can’t think about my health, I’m always thinking about where I’m going to stay [that night]. (32-year-old male)

#### Substance-use triggers

Several participants mentioned their discomfort with the ‘PEH-friendly’ GP clinic catering to active substance users. These participants were ex-IVDUs (intravenous drug users) and struggled with the sight of active users in the reception area. One woman, an ex-IVDU who was excluded from the final sample, specifically refused to continue speaking to A.H. after hearing that our research related to the specialist GP, describing it as ‘full of smackheads’ and a place she would ‘never go’.

The [substance-]users ruin it for everyone else. (52-year-old male)

I’m not someone that likes public places as it is, let alone with druggies around me… That’s not my life anymore. (52-year-old male)

#### Technological barriers in accessing healthcare

All participants noted that if they did not have a phone or access to one at day facilities, accessing mainstream NHS services would be nearly impossible.

I think there’s too much emphasis on booking online or on the phone, a lot of the time homeless people can’t do that. (56-year-old male)

We’ve become too dependent on technology…It prevents homeless people from getting medical care. (56-year-old male)

Observational and interview data showed that most PEH service users interviewed in the day facilities were there to gain communications access, whether via internet on the laptops provided, available handheld phones, free Wi-Fi on personal mobile phones, or information from staff members. PEH experienced considerable social and technological exclusion that could worsen their healthcare outcomes. Even those participants with mobile phones often could not afford data and had to rely on free Wi-Fi networks. All facilities attempted to mitigate this, but participants who wanted increased digital healthcare were a small minority. Few expressed sufficient confidence in their understanding of the health system to successfully navigate digital healthcare services.

### More signposting and outreach to improve healthcare access

Throughout the data collection period, participants and staff consistently mentioned confusion about available ‘PEH-friendly’ GP clinic services. Participants were predominantly aware that it was a GP practice but were unaware of drop-in times, how to register, and where to find the outreach van on which days. Most were unaware that the outreach van was part of its services. In all day facilities visited, few ‘PEH-friendly’ GP clinic leaflets, posters, or signage were observable and those present were small and easily missed. When asked if they had heard of the ‘PEH-friendly’ GP clinic, 12 participants answered ‘yes’, while 8 had not. However, of these eight, six had used outreach van services without knowing they were linked to the ‘PEH-friendly’ GP clinic.

**Figure 1.**
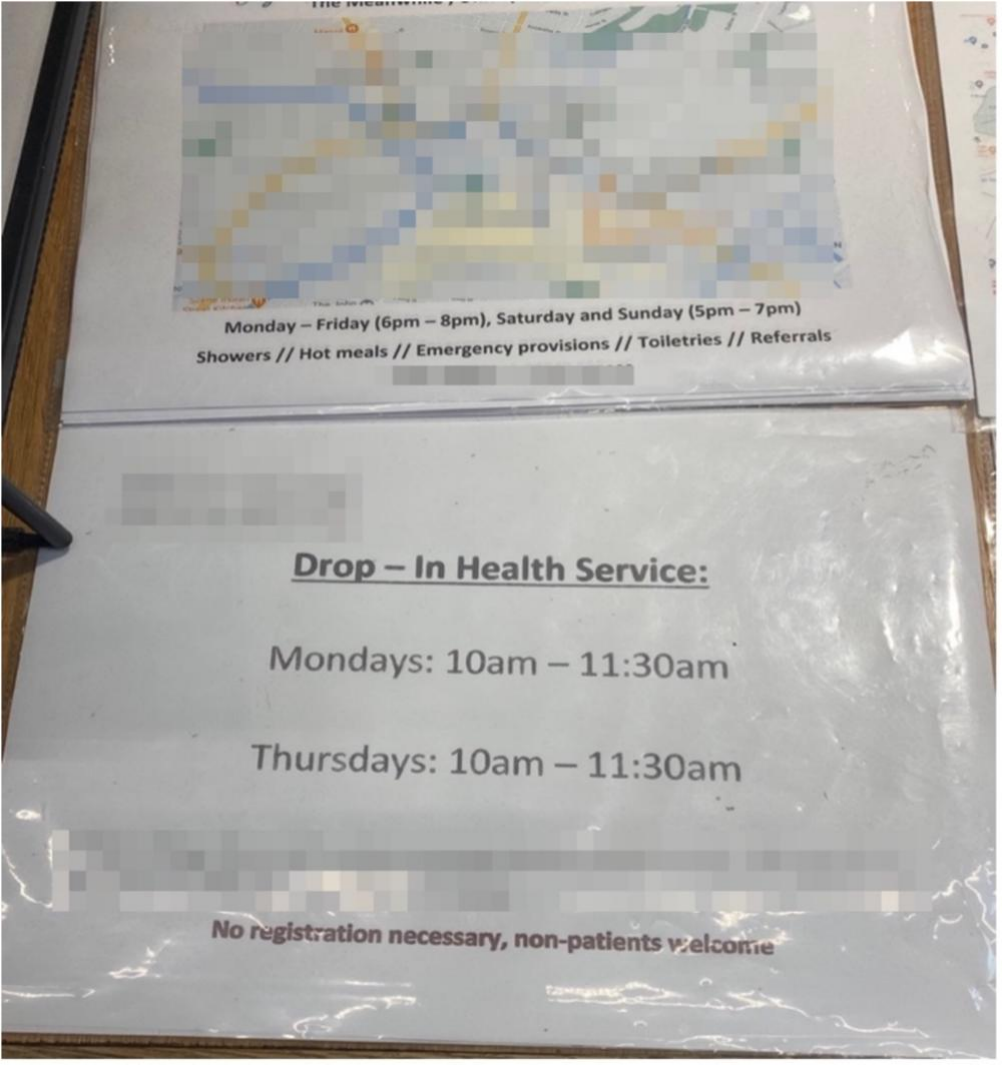
Signage indicating partially accurate location of a ‘PEH-friendly’ facility, opening hours, and services offered (Source: A.H. taken 28 April 2023).

Figure 1 provides an example of ‘PEH-friendly’ GP clinic signage at Facility-2, found in the corner of the reception desk. It was made by facility staff and provided incorrect information (i.e. no drop-in clinics were available on Mondays or Thursdays). When asked about this, service users were unaware of what the sign showed but stated how frustrating it would be if they were to go during those times and find the ‘PEH-friendly’ GP clinic closed.

**Figure 2.**
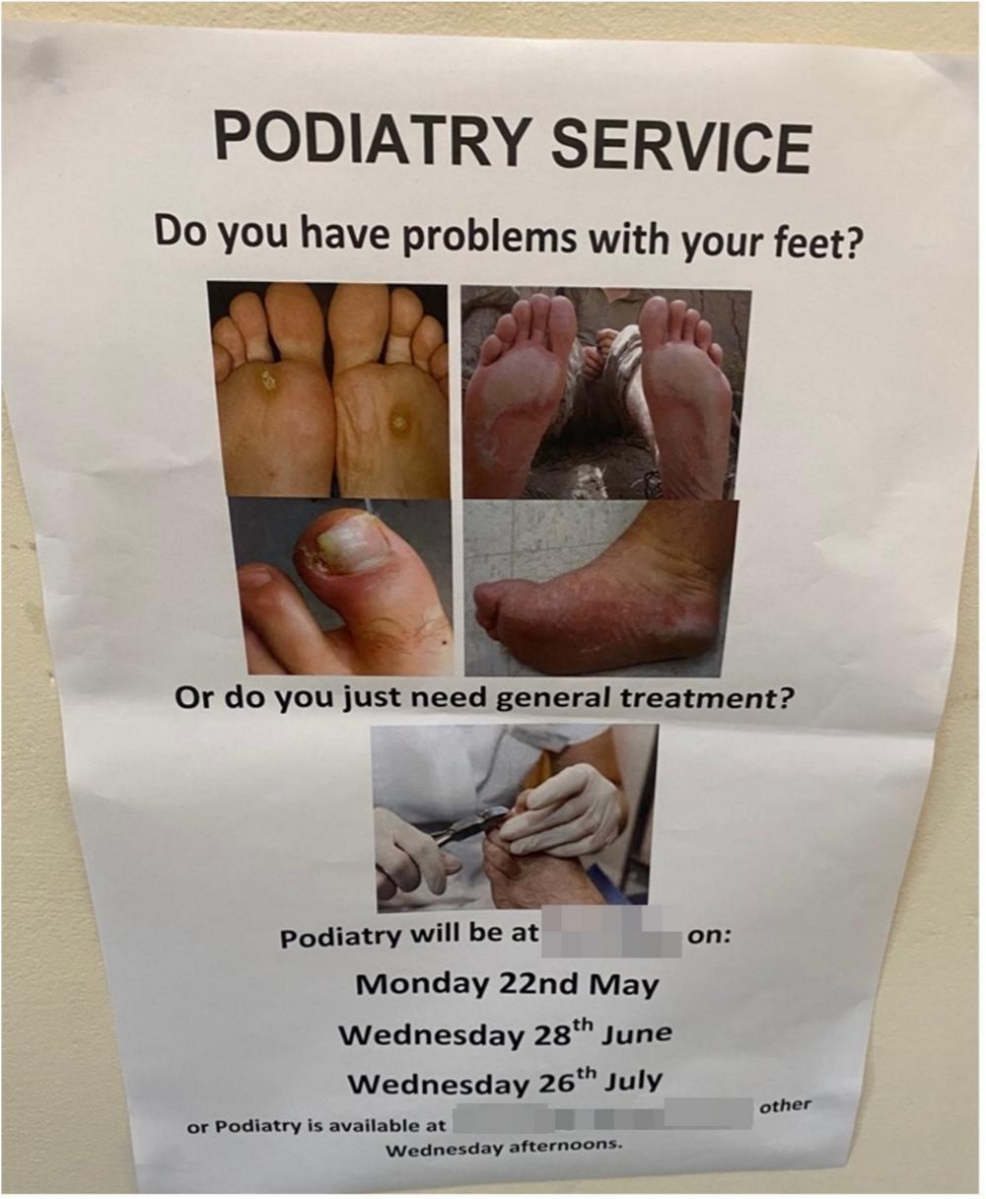
Signage with information on podiatry services offered at a homelessness day facility (Source: A.H., photo taken 9 May 2023).

Figure 2 shows a further example of signage at Facility-3 and Facility-4, both displaying minimal information on what the ‘PEH-friendly’ GP clinic was and where potential service users should go for care. Many participants requested a large poster at each day centre to explain all necessary ‘PEH-friendly’ GP clinic information (e.g. outreach van timetable, opening hours, clinic address, explanation of how to register).

Participants consistently identified travel to the ‘PEH-friendly’ GP clinic as a barrier. Participants who were comfortable walking there and knew where it was or had a smartphone that could provide directions still expressed concern for those not in the same position. Those with injuries (e.g. diabetic foot infections), literacy issues or unable to understand a map, or without mobile phone data were disadvantaged in physically accessing necessary healthcare. Relatedly, participants expressed overwhelmingly positive feedback for the ‘PEH-friendly’ GP outreach van service. All said that such clinical outreach, cutting out difficulties of travel and registration, was invaluable. All participants praised the healthcare staff for providing such a service and attempting to remove some of the known barriers to healthcare access for PEH.

[Nurse’s name] helped dress my wounds after I’d been stabbed a few years back… It makes me emotional to think about. (56-year-old male)

I know people who come here just for the van. (56-year-old male)

[Outreach van nurse] is really down to earth and approachable. (52-year-old female)

Continuity of care was praised in relation to outreach van services, as all participants expressed greater comfort seeing the same nurses each week and developing a trusting rapport with them.

## DISCUSSION

### Key findings

This initial exploration of ‘PEH-friendly’ and mainstream GP services provision from the perspectives of PEH as potential service users provides insight on the context, lived experiences, and perspectives of PEH in relation to health services access. Major concerns PEH identified related to effective communication, health system navigation, information/signage, and outreach, aligning with the literature on PEH and primary healthcare access (Sachs-Ericsson *et al*. 1999, O'Toole *et al*. 2008, Hudson *et al*. 2010, Campbell *et al*. 2015, Elwell-Sutton *et al*. 2017, Gunner *et al*. 2019, Bedmar *et al*. 2022, McNeill *et al*. 2022, Paradis-Gagné *et al*. 2023, Perkin *et al*. 2023). Exploring these perspectives also offers a lens into the changing realities of the UK welfare state at the point of primary healthcare access, exposing fault lines of healthcare reforms and consequent experiences for vulnerable service users (Hyde *et al*. 2016, Rafighi *et al*. 2016, Stafford and Wood 2017, Stuckler *et al*. 2017, Hernandez 2021). Study findings contribute to the literature on PEH experiences and can help inform policy discussions on improving healthcare delivery.

#### Effective communication

PEH described challenges in effectively communicating their health needs, whether due to language or limited health literacy, which could hinder their access to appropriate healthcare services (Robinson 2018, Paradis-Gagné *et al*. 2023). Participants specifically described English language and technological barriers preventing them from feeling empowered to seek healthcare. Similar concerns about technological barriers have been raised for elderly patients in developing and maintaining sufficient digital literacy to access healthcare. As digital technologies and interfaces are rapidly and continually evolving, maintaining digital literacy requires constant updates and effort (Pangbourne *et al*. 2010, Kristiansen *et al*. 2023). This indicates a shared need for communication and health literacy initiatives to ensure that PEH (and elderly healthcare seekers) are not unintentionally further disadvantaged and can understand how to access and use services.

The Equality Act 2010 imposes legal obligations on the NHS to address such access inequalities, and primary care guidelines emphasize patient rights to equal access regardless of language or communication barriers (Whitaker *et al*. 2021). Doctors’ individual responsibility for understanding and implementing patients’ communication preferences and maintaining clear informed consent is also highlighted by the General Medical Council (GMC 2025). As service users with English language difficulties are known to experience primary care access barriers and thus fewer positive health outcomes, the interpretation and translation framework agreement promotes effective communication between patients and clinicians through face-to-face, telephone, and video interpreting and the NHS Language Line is available 24/7 for people contacting emergency services who have concerns about communicating in English (Whitaker *et al*. 2021, NHS24 2023). However, most participants born outside the UK were unaware of these services, suggesting that more efforts are needed to promote them or determine and address the barriers to their use.

#### Health system navigation

PEH and other marginalized individuals (e.g. those with insecure immigration status) often struggle to navigate the complex NHS system, which requires understanding enrolment and referral processes, accessing appropriate services, diagnoses, and recommended treatments, and following up appointments (Woodward *et al*. 2014, Pollard and Howard 2021). Research has documented the challenges that PEH as service users experience in accessing healthcare due to limited health literacy or support with navigation processes (Poduval *et al*. 2015, Rafighi *et al*. 2016, Gunner *et al*. 2019). Participant concerns regarding this are consistent with UK literature, emphasizing the need for clearer pathways to care and streamlining of processes where feasible (Elwell-Sutton *et al*. 2017, Batchelor and Kingsland 2020, McNeill *et al*. 2022). The literature further emphasizes the importance of trusted community partners, such as the outreach van staff, to disseminate information and promote healthcare services access.

#### Signposting and outreach

Effective and accurate signage is a basic way of ensuring stronger care pathways and crucial in informing PEH as service users about dedicated services. Participant concerns and observations regarding inadequate ‘PEH-friendly’ GP clinic signage are thus concerning but also align with the literature highlighting the need for targeted and effective communications to reach PEH (McNeill *et al*. 2022, Perkin *et al*. 2023). Our observations indicated that nobody appeared to be specifically responsible for advertising or updating signage and thus due to competing priorities it was often neglected. Participants suggested useful improvements to signage, and new posters could be reproduced for clinical staff also (e.g. for postdischarge and A&E) so clinicians can also connect PEH to ‘PEH-friendly’ primary care services. While better signage could still exclude those with literacy issues, if posters were updated and posted prominently at day facilities, staff would also be better able to explain services to potential users. Additionally, local authorities could support health promotion by developing a simple map with symbols for essential local services needed by PEH (e.g. healthcare outreach, hospital, food, day facilities and night shelters, housing department, police station, library or other safe spaces for warmth) that could overcome language barriers (Fordham 2012). Providing photos and summary professional information for staff would also align with trauma-informed care (TIC) practices (Hopper *et al*. 2010, Roberts *et al*. 2019).

Participants described challenges in accessing healthcare services due to local travel barriers, including a lack of transportation, finances, and distance. This is supported by the literature on the benefits of outreach services for vulnerable populations in improving healthcare services access and use (Moss *et al*. 2023, Perkin *et al*. 2023). Participant concerns expressed, and identified in the PEH healthcare access literature, indicate the value of mobile and community-based services (e.g. ‘PEH-friendly’ GP outreach van) and flexible delivery models to ensure equitable access (McNeill *et al*. 2022, Paradis-Gagné *et al*. 2023). A.H. presented the study findings to senior management at the ‘PEH-friendly’ GP to help inform quality improvement initiatives in communications and outreach.

### Implications for policy, practice, and further research

Engaging with health equity conceptualizations, particularly SSDH and TIC, can significantly enhance primary health services access and care for PEH (Hopper *et al*. 2010, Stafford and Wood 2017, Roberts *et al*. 2019). SSDH help conceptualize the underlying factors influencing health outcomes, including access to healthcare, for PEH in the UK. These refer to economic, political, and sociocultural factors shaping health outcomes (Crear-Perry *et al*. 2021). In the context of homelessness and NHS access, this recognizes that existing housing policies, income inequality, and support services directly affect PEH health and access to healthcare. SSDH influence health inequalities, with common contributing factors including income, job insecurity, housing, social inclusion, and education. Several of these affect PEH disproportionately more than domiciled populations and affect health outcomes (Campbell *et al*. 2015). Addressing SSDH requires systemic policy and cultural changes to reduce health inequities (Crear-Perry *et al*. 2021).

Most day facilities increasingly emphasize TIC to improve PEH engagement. TIC describes ‘understanding, anticipating, and responding to the issues, expectations, and special needs that a person who has been victimized may have in a particular setting or service’ (Roberts *et al*. 2019). PEH have often experienced traumas, such as neglect, childhood sexual abuse, or community violence (Hopper *et al*. 2010). Homelessness can itself be traumatic and many PEH experience psychiatric issues including depression, addiction, and severe mental disorders, and many are susceptible to revictimization and further social exclusion, lack of resources, and distrust of services (Hopper *et al*. 2010). There is thus growing recognition that TIC could be of benefit in service provision for PEH, but implementation within homelessness services remains limited due to limited research, models to emulate, or collaboration between programmes (Roberts *et al*. 2019).

Particular care must also be must be given to the training and support of healthcare practitioners working with PEH, ensuring that they have the necessary skills and institutional support to meet the challenges of caring for this patient group (Perez *et al*. 2024). For example, the use of medical terms might intimidate or cause discomfort for PEH who may not be most familiar with such technical language; this could discourage and deter them from accessing healthcare services when they need them. Cultivating effective and sensitive communication skills is thus essential and requires further research to develop and deploy appropriate training material for healthcare providers. Without adequate support, healthcare providers working with marginalized populations such as PEH are at heightened risk of vicarious trauma and moral injury, which can have severe repercussions for their own well-being (Waegemakers Schiff and Lane 2019, Čartolovni *et al*. 2021).

In Western countries particularly, healthcare use by PEH is characterized by reliance on unplanned or emergency secondary services, frequently due to limited access to primary healthcare services. In England, it has been estimated that PEH use A&E five to seven times more than the general population (Moss *et al*. 2023). O’Carroll and Wainwright (2019) found that PEH often delayed accessing treatment, despite this poorer health profile, or avoided health services altogether resulting in high burdens of untreated health conditions. PEH may leave hospital before being formally discharged, fail to finish their full treatment course, or miss outpatient appointments for a variety of reasons from felt stigma to competing priorities/unavoidable life events. They may thus come to be seen as ‘revolving door patients’ by healthcare providers, potentially straining the provider–patient relationship and having unintended detrimental effects on the quality of care delivery, service user satisfaction, and individual health outcomes (Shaw 2004). At a health systems level, such noncompliance can contribute significantly to raising healthcare costs (Cleemput and Kesteloot 2002). Therefore, health professionals and health policymakers could considerably improve understanding of their specific health needs and longer-term provision of better health services for PEH in the UK and elsewhere.

### Limitations

Several study limitations should be considered. First, this evaluation took place over nine weeks and was unfunded. This relatively short time period and inability to provide small thank you tokens for participants likely constrained the depth and rigour of data collection. Second, diverse perspectives of PEH were not equally represented. The small number of non-UK-born participants (i.e. six) meant that detailed differentiation in terms of migrant trajectory (e.g. asylum seekers, refugees, trafficked) was not feasible. Women’s perspectives were also under-represented (i.e. only three participants were women). Third, this study relied on qualitative interpretivist methods, and future mixed-methods approaches can provide a more holistic analysis of services provision and access.

## CONCLUSION

This qualitative study explores the experiences and perspectives of PEH in accessing and engaging with health services. The findings indicate that specialized PEH GP services are valued but pathways to care should be clarified, and communication strategies and advertising can be further developed to maintain or increase outreach services and ensure that PEH are better able to access needed services. Gatekeeping behaviours and improving perceived safety for former substance users need to be considered. In addition, there needs to be channels for monitoring and evaluation to track and measure the impact of introduced changes and harness insights from the data to inform further changes. Lessons for mainstream NHS providers include considering how SSDH and trauma-informed approaches can help them to provide better services and access opportunities for marginalized populations.

## Data Availability

All relevant data based on the field notes that A.H. wrote during the study period have been included in the manuscript.
